# University of the City of New York

**Published:** 1850-09

**Authors:** 


*University of the City of New York.*—The Chair of Practice in this Institution has been filled by the appointment of Prof. Elisha Bartlett, of Louisville, Ky., who has signified his acceptance. As a writer and teacher, Prof. B. has few, if any superiors, and the University of the City of New York is fortunate in securing his services. The Chair of Surgery is not yet filled. It is understood that Dr. Mott's separation from the Institution is to be final. Since this article was in type, we learn that Prof. Gross, of Louisville, has been invited to fill the Chair of Surgery, and has accepted.

